# Editorial: The nutritional and health benefits of *Vaccinium* berries

**DOI:** 10.3389/fnut.2025.1561116

**Published:** 2025-02-17

**Authors:** Paulina M. Nowaczyk, Patrick Turck, Małgorzata Jamka

**Affiliations:** ^1^Department of Sports Dietetics, Poznan University of Physical Education, Poznań, Poland; ^2^Department of Physiology, Federal University of Rio Grande do Sul, Porto Alegre, Brazil; ^3^Department of Pediatric Gastroenterology and Metabolic Diseases, Poznan University of Medical Sciences, Poznań, Poland

**Keywords:** *Vaccinium*, flavonoids, anthocyanin, cardiovascular diseases, back pain, cognitive performance, gut microbiota

This Research Topic summarizes the current state of knowledge on the health benefits of selected *Vaccinium* berries (Stull et al.), explores the associations between dietary flavonoid intake and urinary health (Lin et al.) or low back pain incidence (Zhou et al.), as well as indicates novel directions of studies on *Vaccinium* berries, e.g., effects of berries supplementation on cognitive function and gut microbiota (Huang et al.), and the possibilities of enhancing the pro-health benefits of blueberry pomace *via* fermentation (Tian et al.).

Previous research on the pro-health properties of *Vaccinium* berries (with blueberries being at the forefront) and their bioactive compounds has focused on cardiovascular health, pre-diabetes and type 2 diabetes, or brain health and cognitive function (Stull et al.). The perspective literature review by Stull et al. indicated that consuming ≥1–3 servings/week of blueberry may result in reduced risk of myocardial infarction, hypertension, or coronary heart disease (incidence and mortality), effectively lowering total and low-density lipoprotein cholesterol, selected inflammatory markers (i.e., C-reactive protein), and improving vascular function (i.e., endothelial function and arterial stiffness). It is strongly suggested that anthocyanins and their metabolites are key mediators of blueberries' vascular-protective effects. Meanwhile, habitual blueberry intake of ≥2 servings/week may be associated with a lower risk of type 2 diabetes (Stull et al.). Nonetheless, the current body of evidence on blueberry consumption and glucose regulation and insulin resistance in adults yields mixed results, with individuals with normal or near-normal blood glucose concentration only experiencing marginal or negligible improvements in glycemia after blueberry consumption (Stull et al.). Higher berries intake has been associated with a slower rate of cognitive decline, a higher probability of healthy aging with the absence of major chronic diseases and physical and cognitive disability, and a lower risk of Parkinson's disease (Stull et al.). Thus, according to Stull et al., ≥1–2 servings/week of blueberry is recommended to enhance cognitive performance in the contexts of aging and cognitive impairment resulting from brain injury or metabolic and vascular disturbances.

Less-known and less obvious pro-health properties of berries are related to the risk and treatment of urinary tract disorders (Lin et al.) or chronic pain (Zhou et al.). Two articles (Lin et al., Zhou et al.) in the Research Topic utilized data from the National Health and Nutrition Examination Survey (NHANES). According to the literature, 70%−80% of individuals in the general population are likely to experience low back pain at some point in their lives (1, 2). Zhou et al. involved data on 3,136 adults (aged >20 years) participating in NHANES 2009–2010 to explore the association between flavonoid intake and chronic low back pain (CLBP). The findings indicated the modest inverse associations between total flavonoid intake and the likelihood of CLBP. The risk of CLBP was about 26% lower among individuals in the highest tertile group flavonoid intake (>170 mg flavonoid per day) than those in the lowest tertiles (≤ 38 mg flavonoid per day) after adjusting for covariates such as age, gender, poverty income ratio, arthritis, depression, sleep disorder, or use of analgesics. Moreover, individuals who may particularly benefit from flavonoid consumption are those aged ≥45 years with sedentary behaviors and comorbidities such as arthritis, depression, and sleep disorders (Zhou et al.).

Overactive bladder (OAB) is a syndrome marked by urgent urination and night-time voiding, with or without urgent urinary incontinence, in the absence of urinary tract infection or other discernible pathological conditions (3). According to the European Prospective Investigation into Cancer and Nutrition study, the overall prevalence of OAB is 11.8% (4), while in Asian nations like China and South Korea, the prevalence is estimated to be around 20.8% (5). Based on the data of 13,063 American adults participating in NHANES 2007–2008, 2009–2010, and 2017–2018, Lin et al. searched for the association between dietary intake of total and subclass flavonoid and the risk of OAB. Interestingly, after adjusting for confounding factors, the third quartile of consumption of anthocyanidin (11.07–756.1 mg/day) and the second quartile of consumption of flavone (2.06–11.07 mg/day) was significantly associated with the reduced risk of OAB, while total flavonoid consumption was not significantly correlated with the risk of OAB (Lin et al.).

Huang et al. revealed that 18 weeks of feeding C57Bl/6J male mice with high-fat (HF) diet (60% energy from fat) enriched with a mix of freeze–dried powder (20% dry weight basis) of a Nordic berries (lingonberries, bilberries) and red grape juice resulted in improved indicators of spatial memory, increased concentration of the inflammation modifying interleukine-10 cytokine in hippocampal extracts, as well as in significantly greater cecal microbial diversity, as measured by the Shannon diversity index and total operational taxonomic unit (OTU) richness. The HF diet supplemented with berries resulted in a strong trend of higher total OTU richness and significantly increased the relative abundance of *Akkermansia muciniphila* associated with protective effects on cognitive decline (Huang et al.).

Despite the natural and specific abundance of bioactive compounds in *Vaccinium* berries, food science, and the food industry are seeking to further improve the pro-health properties of these fruits. Tian et al. investigated the effect of solid-state fermentation (SSF) of blueberry pomace utilizing three fungi strains and three lactic acid bacterial strains on polyphenol profiles, antioxidant capacities, and bioaccessibility. Ten phenolic acids and six flavonoids were increased in blueberry pomace fermented by *Lactobacillus acidophilus*. A similar tendency was observed after fermentation by *Aspergillus niger* (AN) and *Lactobacillus plantarum*, where the concentration of eight phenolic acids and five flavonoids was enhanced. The anthocyanin decreased after fermentation (apart from fermentation by AN). However, all the evaluated antioxidant activity markers increased, and the bioaccessibility of polyphenolic compounds assessed *via* simulated gastrointestinal digestion increased (Tian et al.). Thus, the polyphenolic compounds and their bioaccessibility in blueberry pomace could be further improved by SSF utilizing appropriate microbial strains.

Despite the promising results on the multidirectional pro-health properties of *Vaccinium* berries and their selected phenolic compounds, numerous research gaps remain to be resolved. First, there is too little evidence from long-term randomized controlled trials on humans, covering various population groups and health domains/conditions. The mechanisms of the *in vivo* action of *Vaccinium* berries have not been elucidated and little is known about the actual bioavailability of phenolic compounds and the factors affecting bioavailability. Furthermore, the effective and safe doses and standardized recommended servings of *Vaccinium* berries (fresh fruit, freeze-dried powders, and supplements) or their bioactive compounds (supplements) must be established.
